# Role of cfDNA and ctDNA to improve the risk stratification and the disease follow-up in patients with endometrial cancer: towards the clinical application

**DOI:** 10.1186/s13046-024-03158-w

**Published:** 2024-09-20

**Authors:** Carlos Casas-Arozamena, Ana Vilar, Juan Cueva, Efigenia Arias, Victoria Sampayo, Eva Diaz, Sara S Oltra, Cristian Pablo Moiola, Silvia Cabrera, Alexandra Cortegoso, Teresa Curiel, Alicia Abalo, Mónica Pamies Serrano, Santiago Domingo, Pablo Padilla-Iserte, Marta Arnaez de la Cruz, Alicia Hernández, Virginia García-Pineda, Juan Ruiz-Bañobre, Rafael López, Xavier Matias-Guiu, Eva Colás, Antonio Gil-Moreno, Miguel Abal, Gema Moreno-Bueno, Laura Muinelo-Romay

**Affiliations:** 1 Translational Medical Oncology Group (Oncomet), Health Research Institute of Santiago de Compostela (IDIS), University Hospital of Santiago de Compostela (SERGAS), Trav. Choupana s/n, Santiago de Compostela, 15706 Spain; 2 University of Santiago de Compostela (USC), Praza do Obradoiro, 0, Santiago de Compostela, 15705 Spain; 3 Centro de Investigación Biomédica en Red de Cáncer (CIBERONC), Madrid, Spain; 4 Department of Gynecology, University Hospital of Santiago de Compostela (SERGAS), Trav. Choupana s/n, Santiago de Compostela, 15706 Spain; 5 Department of Medical Oncology, University Clinical Hospital of Santiago de Compostela, University of Santiago de Compostela (USC), Santiago de Compostela, 15706 Spain; 6 MD Anderson Cancer Center Foundation, C/Gómez Hemans 2, Madrid, 28033 Spain; 7 Department of Gynecologic Oncology, Biomedical Research Group in Gynecology, Vall Hebron Research Institute (VHIR), Universitat Autónoma de Barcelona, 119-129 Pg. Vall d’Hebron, Barcelona, 08035 Spain; 8 Department of Basic Sciences, Faculty of Health Sciences at Manresa, University of Vic – Central University of Catalonia (UVic-UCC), Av. Universitària, 4-6, Manresa, 08242 Spain; 9 Department of Gynecologic Oncology, La Fe University and Polytechnic Hospital, Valencia, Spain; 10 Department of Gynecology, University Hospital “La Paz”, Madrid, Spain; 11 Department of Pathology, Instituto de Investigación Biomédica de Bellvitge, Centro de Investigación Biomédica en Red de Cáncer, Hospital U de Bellvitge and Hospital U Arnau de Vilanova, Universities of Lleida and Barcelona, Institut de Recerca Biomèdica de Lleida, Barcelona, Spain; 12 Instituto de Investigaciones Biomedicas “Sols-Morreale” CSIC-UAM, C/Arturo Dupurier 4, Madrid, 28029 Spain

**Keywords:** Liquid Biopsy, Endometrial cancer, Blood biomarkers, Prognostic biomarkers, Tumour kinetics

## Abstract

**Background:**

There has been a rise in endometrial cancer (EC) incidence leading to increased mortality. To counter this trend, improving the stratification of post-surgery recurrence risk and anticipating disease relapse and treatment resistance is essential. Liquid biopsy analyses offer a promising tool for these clinical challenges, though the best strategy for applying them in EC must be defined. This study was designed to determine the value of cfDNA/ctDNA monitoring in improving the clinical management of patients with localized and recurrent disease.

**Methods:**

Plasma samples and uterine aspirates (UA) from 198 EC patients were collected at surgery and over time. The genetic landscape of UAs was characterized using targeted sequencing. Total cfDNA was analyzed for ctDNA presence based on the UA mutational profile.

**Results:**

High cfDNA levels and detectable ctDNA at baseline correlated with poor prognosis for DFS (p-value < 0.0001; HR = 9.25) and DSS (p-value < 0.0001; HR = 11.20). This remained clinically significant when stratifying tumors by histopathological risk factors. Of note, cfDNA/ctDNA analyses discriminated patients with early post-surgery relapse and the ctDNA kinetics served to identify patients undergoing relapse before any clinical evidence emerged.

**Conclusions:**

This is the most comprehensive study on cfDNA/ctDNA characterization in EC, demonstrating its value in improving risk stratification and anticipating disease relapse in patients with localized disease. CtDNA kinetics assessment complements current strategies to monitor the disease evolution and the treatment response. Therefore, implementing cfDNA/ctDNA monitoring in clinical routines offers a unique opportunity to improve EC management.

**Translational relevance:**

The study demonstrates that high levels of cfDNA and detectable ctDNA at baseline are strong indicators of poor prognosis. This enables more accurate risk stratification beyond traditional histopathological factors, allowing clinicians to identify high-risk patients who may benefit from more aggressive treatment and closer monitoring. Moreover, longitudinal analysis of cfDNA/ctDNA can detect disease recurrence months before clinical symptoms or imaging evidence appear. This early warning system offers a significant advantage in clinical practice, providing a window of opportunity for early intervention and potentially improving patient outcomes.

**Supplementary Information:**

The online version contains supplementary material available at 10.1186/s13046-024-03158-w.

## Background

In the past years, there has been a rise on the number of patients with advanced endometrial cancers (EC) patients, resulting in an increase on the EC mortality rates [1, 2]. Moreover, currently available therapeutic approaches for EC patients have shown limited efficacy in advanced disease [3]. Approximately 20% of EC patients will develop recurrent disease or distant metastases after primary treatment [1]. The identification of patients at higher risk of recurrence is an urgent unmet clinical need. In fact, for many years the risk stratification has not practically changed and mostly is based on pathological and molecular information. Thus, characteristics such as high-grade, advanced FIGO stage, non-endometrioid histology and the combination of these features with molecular subgroups have refined the prognostic prediction [1, 4–6]. In patients with high-intermediate/high-risk tumours, clinical guidelines recommend adjuvant treatment to eliminate potential residual disease after surgery [5]. However, only a fraction of these patients actually benefit from the adjuvant treatment, as more than 80% are considered as cured after surgery [7]. Thus, more precise tools are required to improve the risk stratification and the selection of patients that should receive adjuvant therapy.

Another key challenge is the sequential therapy regimens during disease evolution. Although EC treatment is evolving and the immunotherapy and targeted therapies are gaining more interest, the most effective treatment combination for each patient at the best moment requires precise follow-up tools to anticipate the emergence of resistance [1, 8]. Tumour heterogeneity and clonal evolution in response to therapy are also key milestones to overcome in order to improve the EC patient management [9, 10]. In fact, the molecular characterization of uterine aspirates (UAs) from EC patients has clearly showed the relevance of minimally invasive samples to capture the genetic heterogeneity present in the primary tumour [9, 10]. Besides, the analysis of circulating biomarkers such as circulating cell-free DNA (cfDNA), allows the dynamic characterization of the tumour with minimal discomfort to the patients and provides valuable real-time information on disease evolution [11–16]. Although few studies have been published focusing on the value of liquid biopsy-based approaches in EC, they have shown promising results [17, 18]. For instance, higher levels of cfDNA were associated with high-risk tumours [19–25]. Moreover, Bolivar et al. showed that high levels of cfDNA correlate to worse disease free survival (DFS) and disease specific survival (DSS) [23]. Regarding the specific tumour-derived cfDNA (ctDNA), our group has reported that approximately 40% of patients with EC have detectable levels of ctDNA at surgery, being patients with higher levels of ctDNA those with a higher risk of recurrence [19, 24, 26].

Considering all this scientific and clinical context and progress towards the application of liquid biopsy in EC, the current study was designed to determine the value of cfDNA/ctDNA to complement the current risk stratification and to assess their capability to anticipate the disease recurrence with the final goal of improving the clinical management of patients with localized and recurrent disease.

## Materials and methods

### Patients’ inclusion and sample collection

A total of 198 patients with EC were recruited between January 2018 and June 2022 at the Gynecology Department, Vall d’Hebron University Hospital (Barcelona, Spain), MD Anderson Cancer Center (Madrid Spain), University Clinical Hospital of Santiago de Compostela (Santiago de Compostela, Spain) and the University Hospital La Fe (Valencia, Spain). This study has followed all ethical recommendations established by the Spanish regulation (Ley de Investigación Orgánica Biomedica, Jul, 14th 2017) and was approved by the ethic committees of the participating institutions. Briefly, patients were prospectively included into the study if the following criteria were met: (a) patients diagnosed with endometrial adenocarcinomas of any histology; (b) patients older than 18 years; (c) patient’s signed informed consent; (d) patients were not receiving antitumoral treatment at the time of sample collection; (e) patients did not have a history of cancer within the last 5 years prior to sample collection, (f) patients who do not have a previous family history of oncology related to the tumor under study.

All UAs were collected at surgery using a Cornier cannula and processed within the first hour after extraction as previously described [26]. Briefly, equal amounts of UA and PBS were mixed in a 1:1 ratio and centrifuged at 2500xg for 20 min at 4ºC, the supernatant and pellet were isolated and stored at -80ºC until use. Peripheral blood samples were collected using CellSave Preservative tubes (Silicon Biosystems Inc, Huntington Valley, USA). Plasma was isolated by a two-step centrifugation (1500xg and 5500xg, respectively) always within the first 48 h after collection. Longitudinal peripheral blood samples were mainly collected every 6 months for 2 years after surgery, and when a recurrence was suspected or confirmed by imaging and/or biopsy. In a subset of 37 patients, blood was also collected 1 month after surgery, in addition to the above follow-up scheme.

### Nucleic acids isolation

DNA and RNA from the UA were obtained using RecoverAll™ Total Nucleic Acid Isolation Kit (Thermo Fisher Scientific, Waltham, MA, USA) following the manufacturer’s conditions. DNA from plasma samples was extracted from 5mL of plasma with the QIAamp DNA Circulating Nucleic Acid Kit (Qiagen, Venlo, Netherlands), according to the manufacturer’s instructions. DNA and RNA from FFPE samples were isolated using the AllPrep DNA/RNA FFPE Kit (Qiagen, Venlo, Netherlands). All DNA samples were quantified using the Qubit Fluorometer (Thermo Fisher Scientific, Waltham, MA, USA) and stored at -20ºC until use.

### CfDNA characterization by ddPCR

For each patient, specific ddPCR assays (90% of the assays were wet-lab validated by Bio-Rad, 10% were custom assays) were designed based on the genomic landscape identified in the UA and run on a QX-200 dPCR system (Bio-Rad, California, USA). ddPCR reactions were performed with 30ng of cfDNA in most cases, in those patients with lower amounts of cfDNA the maximum possible amount was used but never less than 10ng per assay. PCR was performed with the ddPCR Supermix for probes (Bio-Rad, Santa Rosa, CA, USA). The sample was partitioned into a median of 50,000 droplets (across triplicates) in an automated droplet generator (Bio-Rad, CA, USA), according to the manufacturer’s instructions. Emulsified PCR reactions were run on 96-well plates on a C1000 Touch™ thermal cycler (Bio-Rad, CA, USA) according to the manufacturer’s instructions. Plates were read on a Bio-Rad QX-200 droplet reader with Bio-Rad’s QuantaSoft v1.7.4 software to quantify the number of droplets positive for mutant DNA, wild-type DNA, both, and neither. Analysis was performed manually by two independent molecular biologists according to the following guidelines: a minimum of 30,000 positive droplets across wells were required for a valid assay, and a minimum of five, single FAM-positive or HEX-positive droplets with ≤ 2 positive events in the WT control were required to consider samples as mutated. If any events were found on the negative template control or more than two positive events were found on the WT control, the ddPCR was repeated. The blank and detection limits were set at 0.04% [0.01-0.05%] and 0.1% [0.05-0.2%], respectively, for all assays.

### Targeted sequencing of the uterine aspirate

DNA and RNA extracted from UAs were targeted sequenced using the Oncomine Comprehensive Panel v3 (Thermo Fisher, Pleasanton, CA) according to previously published protocols [19, 26]. This panel includes 161 genes categorized by somatic alteration type, including 87 hotspots genes, 43 focal CNV gains, 48 full CDS for DEL mutations, and 51 fusion drivers, covering all the most common mutations found in EC.

In summary, 10 ng of both DNA and cDNA from each UA were utilized for library assembly by multiplex PCR on an AB2720 Thermal Cycle (Life Technologies, Carlsbad, California, USA), adhering to the manufacturer’s instructions. PCR amplification was performed in 18 and 20 cycles. Subsequently, primary primers underwent partial digestion using FuPa reagent (Thermo Fisher, Pleasanton, CA, USA). The Ion P1 Adapter and Ion Xpress Barcode X were employed for amplicon ligation, followed by library purification and quantification using the Ion Library TaqMan Quantitation Kit and ViiA 7 system. Libraries were diluted to match the concentration range of the Escherichia coli DH10B control library standards, with relative concentration determined through qPCR analysis. Template preparation and enrichment were conducted using the Ion S5 XL system. Diluted libraries were combined with template-positive Ion Sphere Particles (ISPs) and Ion S5 enzyme mix for emulsion PCR, followed by enrichment on the Ion OneTouch 2. Targeted mass sequencing was performed on the S5 sequencer (Thermo Fisher, Pleasanton, CA, USA) with six libraries (RNA and DNA) run on 540 chips. Duplicates were analysed for 10% of the samples and yielded consistent results.

For the bioinformatic analyses, alignment to the Hg19 human reference genome and variant calling were executed using Torrent Suite Software v.15.1 (Life Technologies). Variants with a Phred quality score field value < 100 were considered low-quality, while the prediction of genomic variant effects on protein function was performed using the Alamut Visual Plus. Variants predicted as possibly damaging or deleterious were visually inspected with Integrative Genomics Viewer (IGV) v.2.3.40, Broad Institute. Variants with a global minor allele frequency above 0.05 were categorized as single nucleotide polymorphisms and excluded (data from dbSNP, http://www.ncbi.nlm.nih.gov/SNP/). Importantly, when a genetic alteration previously described as a genetic susceptibility variant was identified, it was validated by an alternative sequencing method.

### Statistical analysis

Statistical analysis was conducted in R (R Core Team, 2020) and figures were generated using ggplot2 (122) and GraphPad Prism 8.0 (GraphPad Software, Inc., San Diego, CA, USA). A Cox-proportional hazard model was used to determine the correlation of clinical and experimental variables with clinical outcomes. Wilcoxon’s signed-rank test was used to evaluate statistical differences in non-parametric experimental variables. The Spearman correlation test was performed to determine the relationship between experimental nonparametric variables. The Pearson correlation test was performed to determine the relationship between parametric quantitative experimental and clinical variables. Associations between clinicopathologic features and the experimental variables were examined with the chi-square test (Fisher’s exact test). The RegParallel package [27] was used to stablish the optimal cut-point to determine the cfDNA utility as a predictor of poor clinical outcome. A P-value < 0.05 was set as the level of statistical significance.

## Results

### Clinicopathologic characteristics of the cohort

A total of 198 patients with EC and with at least 6 months of post-surgery follow up have been prospectively included in the study. Clinical characteristics are summarised in Supplementary Table 1. The cohort included patients with endometrioid (76%) and non-endometrioid carcinomas (24%), low (G1-2) and high grade (G3) (58% and 42%, respectively), FIGO stage I-IV (65%, 15%, 15%, 4.7%, respectively) tumours from all TCGA groups (POLE 7,7%, MSI 39%, NSMP 27%, HCN 26%) classified accordingly with the updated ESMO/ESGO consensus [1]. A total of 37 (19%) patients had a relapse with a median DFS of 13.9 months [2.6–49.2]. Twenty-four patients (12%) died of disease, showing the global cohort a median DSS of 19.1 months [8.6–45.7].

### Targeted sequencing of UAs for personalized ctDNA detection

The UAs from all the patients were subjected to NGS using a targeted panel previously used to characterize EC patients [10, 26]. With this strategy we identified pathogenic mutation in 189 of the patients (95.46%) The 10 most frequently mutated genes for SNPs were *PTEN* (54.46%), *PIK3CA* (48.51%), *TP53* (30.69%), *ARID1A* (28.71%), *KRAS* (20.79%), *CTNNB1* (19.31%), *PIK3R1* (17.82%), *FBXW7* (14.36%), *PPP2R1A* (13.37%) and *FGFR2* (9.41%). Focusing on genomic alterations, 19 patients (9.59%) had CNAs. The top 10 altered genes for CNAs are *CCNE1* (4.46%), *ERBB2* (2.48%), *CDK2* (1.98%), *AKT2* (0.99%), *MDM2* (0.99%), *MYC* (0.99%), *PIK3CA* (0.99%), *AR* (0.50%), *AXL* (0.50%) and *CCND3* (0.50%). These data are consistent with the most common alterations of EC described in tissue and UAs samples [26, 28].

**Fig. 1:**
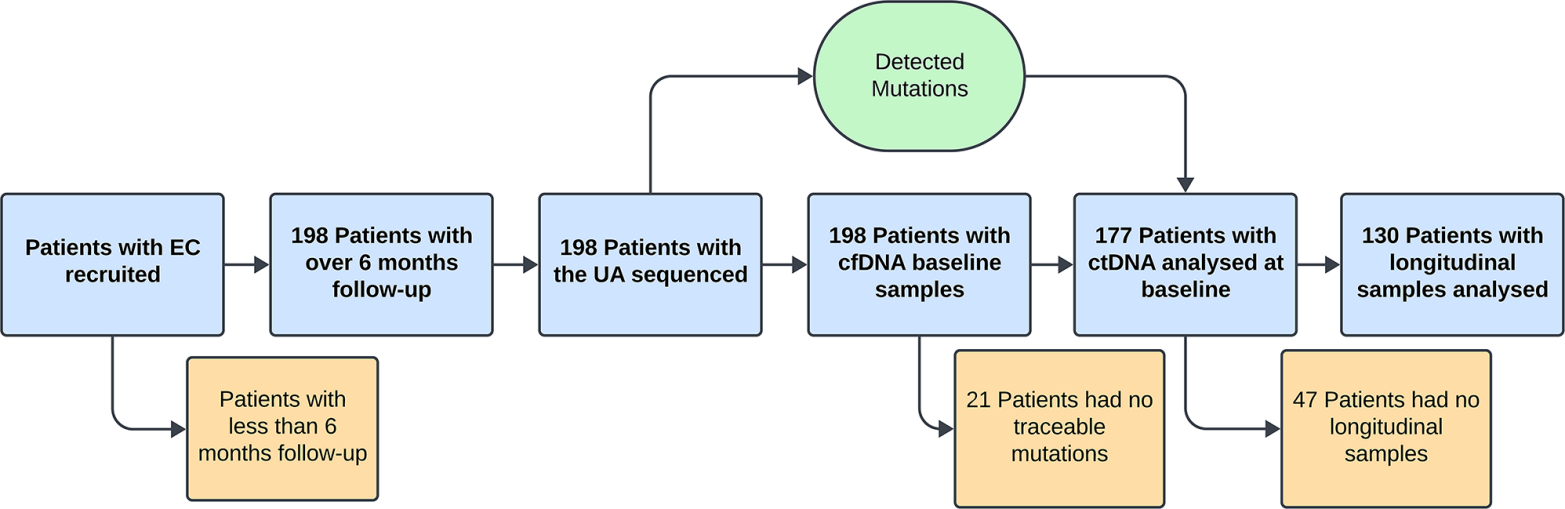
Schematic representation of the cfDNA and ctDNA analyses in the cohort of endometrial cancer patients. Consort plot showing the number of patients excluded at each time point of the study

Based on the genetic alterations found in the UAs, specific ddPCRs were designed to monitor those with the highest allelic frequency. In MSI tumours, a panel of 5 microsatellite markers was also assessed by ddPCR. Overall, we could design effective assays to monitor ctDNA in 177 patients. In addition to the pre-surgical time point, we obtained follow-up blood samples from 130 patients (65.66%) to study the value of longitudinal cfDNA/ctDNA monitoring (Fig. 1).

### Pre-surgery CfDNA levels have independent prognostic value in endometrial cancer patients

**Fig. 2:**
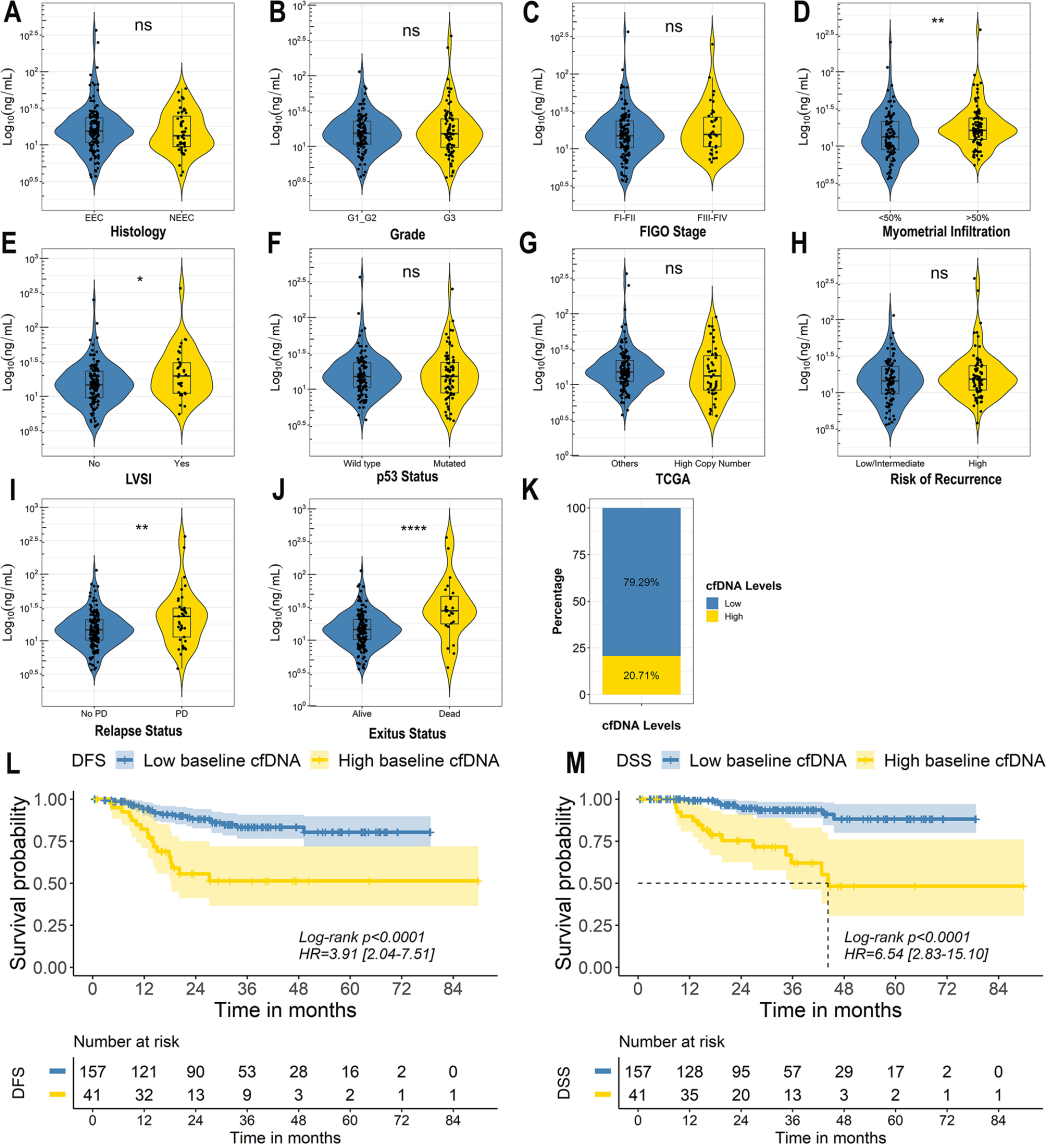
The value of pre-surgery cfDNA in identifying patients with poor clinical outcome. **A-J**. Violin plots of the pre-surgery cfDNA levels (Log10 ng/mL) according to the clinicopathologic variables of the tumours. Statistical significance was evaluated by based on Mann–Whitney U test **p < 0.01. **K**. Classification of the patients as low or high pre-surgery cfDNA based on the optimal cut point (25 ng/mL). **L-M**. Kaplan Meier curves showing DFS (**L**) and DSS (**M**) based on pre-surgery cfDNA levels. Univariate Cox proportional-hazards model was used to estimate HR and log-rank test to report p-value

The total cfDNA concentration was evaluated to determine its association with the pathological findings and risk of recurrence. Total cfDNA was isolated from plasma samples (3-5mL) obtained at the time of tumour resection and quantified using Qubit fluorometry. The total cfDNA concentration at surgery ranged between 3.62 and 366.80 ng/mL with a mean value at 21.94 ng/mL and a median of 15.12 ng/mL. Higher levels of cfDNA correlated with traditional high risk of recurrence markers, although statistically significance was only found for myometrial and lympho vascular infiltration (Mann Whitney test p-value > 0.05) (Fig. 2A-H). Accordingly, significantly higher pre-surgery cfDNA levels were found in patients who showed disease recurrence or died because of disease (Mann Whitney test p-value < 0.01) (Fig. 2I-J). An optimal cut-off at 25ng/mL was determined using on the RegParallel package to group patients into high or low cfDNA levels and to explore the utility of cfDNA as a predictor of clinical outcome. Following this strategy 20.70% (41/198) of patients showed high pre-surgery cfDNA levels (Fig. 2J). These patients had a significantly shorter DFS (Log-rank test p-value < 0.0001; HR = 3.91; 95% CI [2.04–7.51]) and DSS (Log-rank test p-value < 0.0001; HR = 6.54; 95% CI [2.83–15.10]) than those with low levels of pre-surgery cfDNA (Fig. 2L-M, respectively). In addition, multivariant analyses showed that cfDNA levels had independent prognostic value to predict DFS (Log-rank test p-value = 0.008; HR = 2.98; 95% CI [1.35–6.61]) and DSS (Log-rank test p-value < 0.001; HR = 9.13; 95% CI [2.82–29.50]) (Supplementary Table 3). Moreover, the correlation between cfDNA levels and standard blood biomarkers used clinical for follow-up, such as CA-125 or CEA, was analysed in subset of the cohort. Importantly, cfDNA levels did not correlate with CA-125 and CEA levels (Spearman *R* < 0.1) (Supplementary Fig. 1A-C).

No correlation between cfDNA levels and leucocytes count was found, although these blood cells has been reported as the main responsible for the plasma cfDNA content in other tumour types [29]. Furthermore, to ensure that the value of cfDNA and ctDNA were not biased towards tumour size or volume we performed a comparison of cfDNA levels and ctDNA positivity with tumour length (Supplementary Fig. 1D-F, respectively) and with tumour volume (Supplementary Fig. 1E-G, respectively), finding no differences between these variables.

### ctDNA as a minimally invasive prognostic tool in endometrial cancer patients

**Fig. 3:**
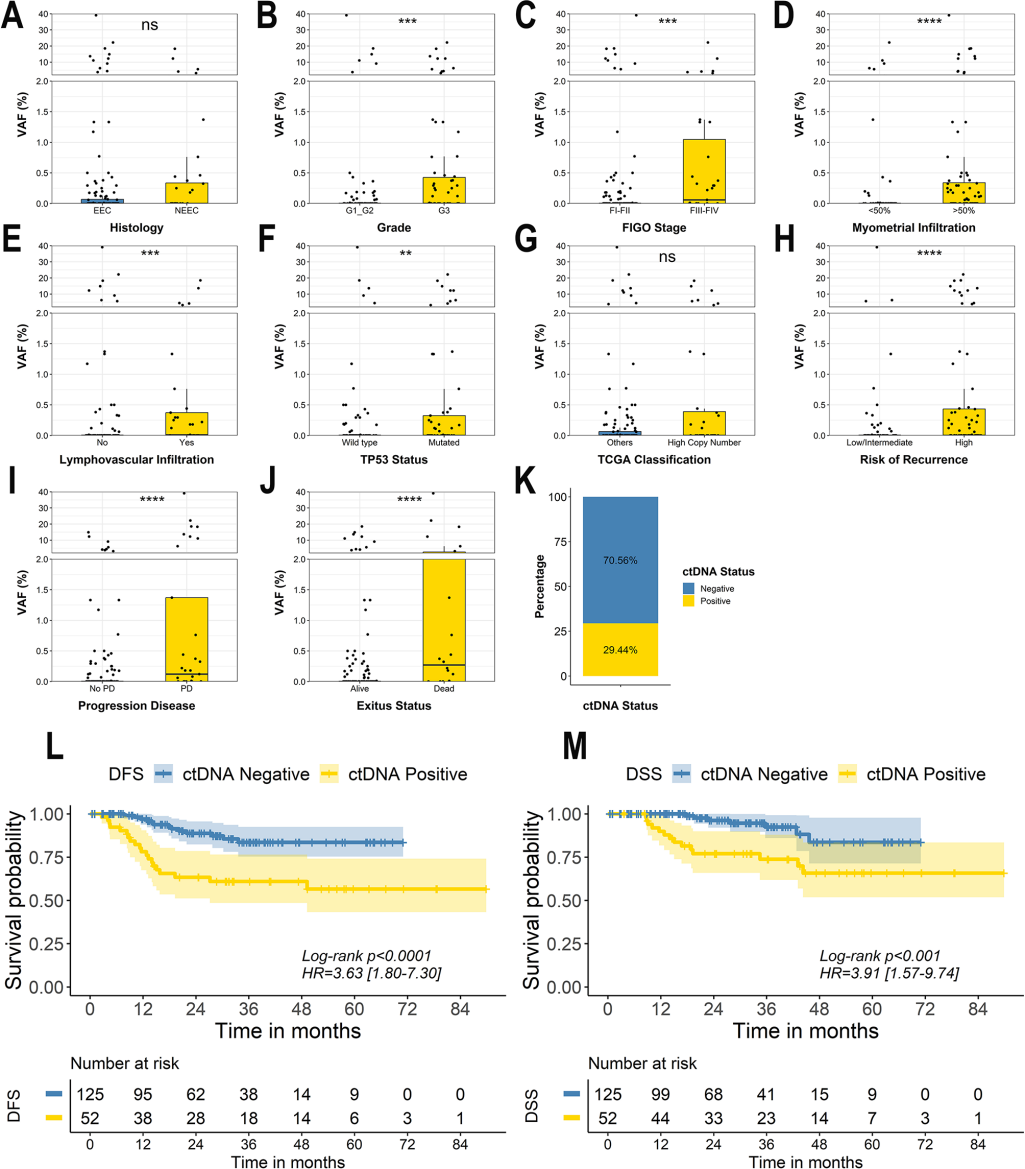
The value of ctDNA analyses in endometrial cancer. **A-J**. Box plots showing the highest variant allelic frequency (VAF %) of the alterations found in the ctDNA accordingly the patient clinical variables. Statistical significance was assessed based on Mann–Whitney U test **p < 0.01, **p < 0.001, ****p < 0.0001. **K**. Percentage of patients with positive and negative ctDNA levels. **L-M**. Kaplan Meier curves showing DFS (**L**) and DSS (**M**) in patients with positive vs. negative levels of ctDNA. Univariate Cox proportional-hazard model was used to estimate HR and log-rank test to report p-value

The levels of ctDNA were determined in a total of 177 patients using personalized ddPCR assays based on the mutational profile found in the UA and 52 (29.38%) of them showed detectable levels of ctDNA (Fig. 3K) with a variant allelic frequency (VAF) in a range from 0.01 to 39.10%, an average of 4.08% and a median of 0.44%. Pre-surgery ctDNA positivity was significantly associated with higher levels of cfDNA (Mann Whitney, p-value < 0.01) (Supplementary Fig. 2A) being this association partially explained by a lower cfDNA input used for the ddPCR in patients with low cfDNA (Supplementary Fig. 2B). However, pre-surgery cfDNA levels and ctDNA VAF were not correlated in those patients with detectable ctDNA (Spearman *R* ≤ 0.2) (Supplementary Fig. 2C-D). Higher detection rates and VAFs were observed in tumours with clinico pathological features of high risk, more specifically in patients with high grade, FIGO III-IV, over 50% myometrial infiltration or LVSI (Mann Whitney, p-value < 0.01) (Fig. 3A-H) (Supplementary Table 2), confirming that higher risk EC tumours shed into circulation higher ctDNA contents.

With the aim to understand if pre-surgery ctDNA can provide additional information to predict the disease prognosis we grouped patients in positive and negative for ctDNA presence and performed survival analyses. Of note, patients with detectable levels of ctDNA showed significant shorter DFS (Log-rank test p-value < 0.001; HR = 3.63; 95% CI [1.80–7.30]) and DSS (Log-rank test p-value < 0.01; HR = 3.91; 95% CI [1.57–9.74]) when compared to patients with undetectable ctDNA (Fig. 3L-M, respectively, Supplementary Table 4).

### Combinatory analysis of cfDNA and ctDNA identifies patients with worst clinical outcomes

Since cfDNA and ctDNA levels independently provide prognostic information, we aimed to explore whether combining both we could improve the identification of patients at higher risk of EC recurrence. To this end, patients were stratified according to cfDNA levels and the presence/absence of ctDNA at surgery. Using this approach 4 groups were set up: ‘Group 1’ cfDNA-low/ctDNA-negative (58.76%); ‘Group 2’ cfDNA-low/ctDNA-positive (20.34%); ‘Group 3’ cfDNA-high/ctDNA-negative (11.86%) and ‘Group 4’ cfDNA-high/ctDNA-positive (9.04%). The clinical characteristics of the patients included in each group are summarised on Supplementary Table 5.

**Fig. 4:**
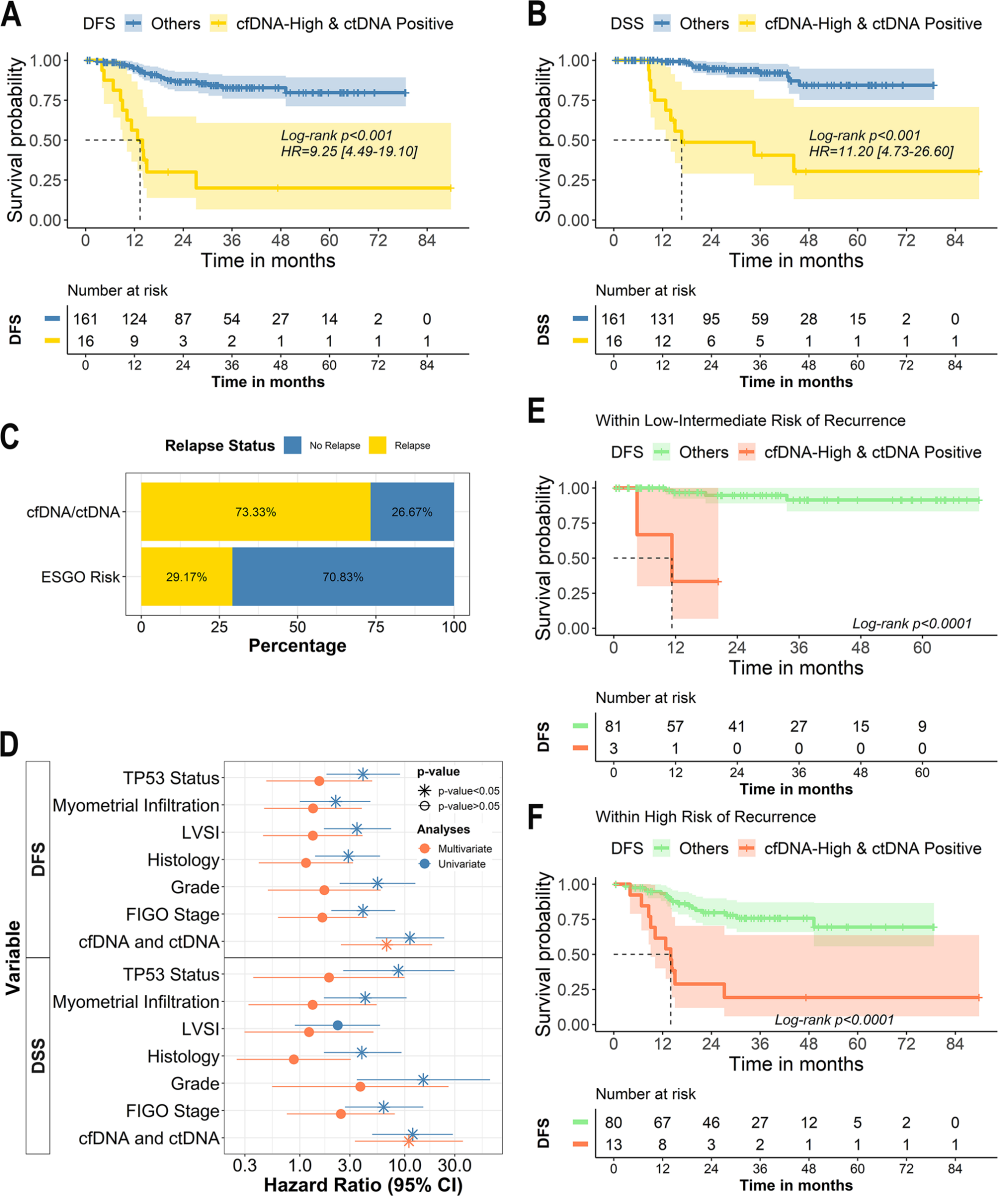
Combined analyses of cfDNA and ctDNA identify the patients with the worst clinical outcome. **A-B.** Kaplan Meier curves showing DFS (**A**) and DSS (**B**) in patients according to the pre-surgery high levels of cfDNA and detectable levels of ctDNA. **C.** Bar plot with the early recurrence status according to the combinatorial approach and the ESGO risk classification **D.** Graphical representation of the univariate (blue) and multivariate (red) Cox proportional-hazard models. P-value > 0.05 is represented with the *symbol. **E-F.** Kaplan-Meier curves showing DFS in patients according to the pre-surgery high levels of cfDNA and detectable levels of ctDNA in patients with low or intermediate (**E**) and high-intermediate or high (**F**) risk of recurrence based on the ESGO-ESTRO-ESP risk stratification

Patients in group 4 (cfDNA-High/ctDNA-positive) had the worst results in terms of DFS (Log-rank test p-value < 0.0001; HR = 9.25; 95% CI [4.49–19.10]) and DSS (Log-rank test p-value < 0.0001; HR = 11.20; 95% CI [4.73–26.60]) when compared to the remaining groups (Fig. 4A-B, respectively). Patients in group 3 (cfDNA-high/ctDNA-negative) showed poorer survival rates than patients in group 1 (cfDNA-low/ctDNA-negative) but similar with the group 2 (cfDNA-low/ctDNA positive) (Supplementary Fig. 3A-B). These results could be associated with the presence of very low ctDNA levels in patients included in group 3 that were not detected due to the limitations of the ddPCR approach, although cfDNA high levels indicate an aggressive disease.

Notably, 75% of the patients included in group 4 (characterized by high cfDNA and ctDNA positivity) had a disease relapse, and 69% of them died during the follow-up as a result of the disease. Of note, 73% these patients had a relapse within the first year after the surgery while only the 30% of tumours classified as high-intermediate and high risk of recurrence, according to the latest ESGO-ESTRO-ESP risk classification, showed an early relapse (Fig. 3C). Importantly, this combinatory approach showed independence over the traditional risk factors and molecular subtype (Fig. 3D). Besides, this combinatory approach remains clinically significant when stratifying patients based on or according to histology, grade or FIGO stage (Supplementary Fig. 3C-F). Importantly, the presence of high levels of pre-surgery cfDNA and ctDNA positivity in patients classified as low or intermediate risk based on ESGO-ESTRO-ESP criteria was associated with a quick relapse in three cases, although most of the patients had a good prognosis (Fig. 4E). And notably, patients classified as high-intermediate/high risk of recurrence based on ESGO-ESTRO-ESP criteria and with high levels of pre-surgery cfDNA and the ctDNA positivity showed a very aggressive disease (Fig. 4F). Therefore, the analysis of liquid biopsy clearly complements the current tools to anticipate disease relapse (Supplementary Fig. 3G-H).

### Combination of risk classification and cfDNA/ctDNA to predict the patients’ outcome

We combined the current risk stratification tools with the risk groups derived from the liquid biopsy analyses. With this strategy we considered a patient in the group of poor prognoses if she has a high-intermediate or high-risk tumour (ESGO-ESTRO-ESP criteria) or high cfDNA/ctDNA positivity at surgery. This approach identified 54.23% (96/177) of the cohort as poor prognoses. With this approach the HRs of the poor prognosis group associated with the DFS (Log-rank test p-value < 0.0001; HR = 7.91; 95% CI [2.39–26.20]) and DSS (Log-rank test p-value < 0.0001; HR = 16.10; 95% CI [2.14–120]) were even more prominent than when analyse this classification strategies independently (Supplementary Fig. 4A-B, respectively). Moreover, 90% and 95% of the patients who underwent disease recurrence and died because of the disease respectively, were classified as high-risk patients thanks to the inclusion of the liquid biopsy in the analysis.

### CfDNA and ctDNA as a monitoring tool for EC

To explore the value of cfDNA and ctDNA monitoring as a surrogate of the EC burden, a total of 372 longitudinal blood samples were analysed in a subset of 130 patients. From these patients, 22 showed disease progression. Significant reduction on the cfDNA levels were found after surgical resection at 1, 6 and 24 months (Wilcoxon signed-rank test, p-value < 0.05, Supplementary Fig. 5A-F), although the dynamics of cfDNA levels in the longitudinal samples did not show value to anticipate the disease reappearance in our cohort of patients.

**Fig. 5:**
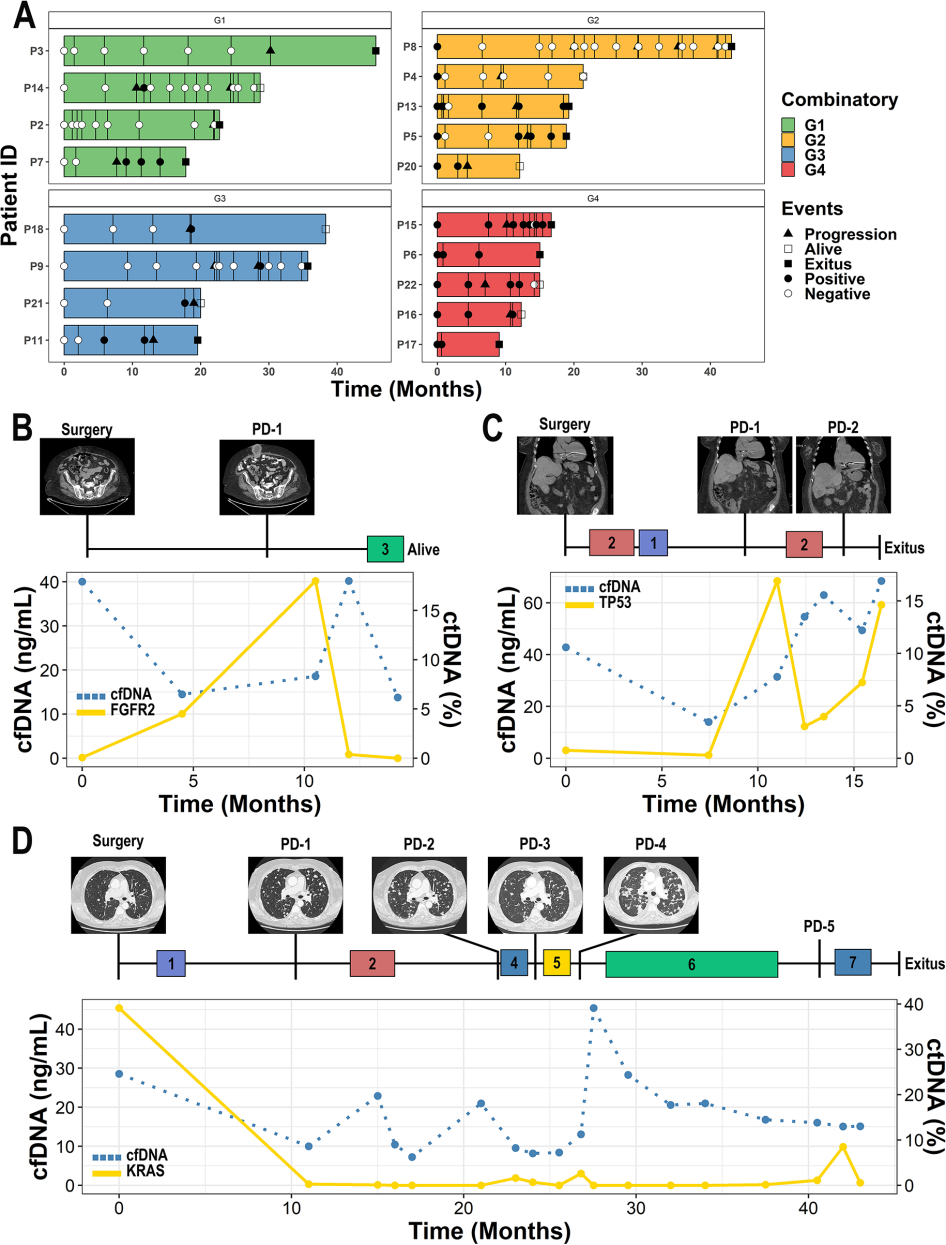
Longitudinal analyses of cfDNA/ctDNA allow for early detection and accurately reflect the disease kinetics. **A.** Swimmer plot of the 18 patients that underwent tumour progression divided based on the combinatorial approach with longitudinal samples collected at least 6 months prior to the relapse (cfDNA and ctDNA). **B-D**. Example figures of the cfDNA (blue dotted line) and ctDNA kinetics (yellow line) in patients with advanced disease. (1) Radiotherapy, (2) Carboplatin-Paclitaxel, (3) Dostarlimab, (4) Exemestane, (5) Doxorrubicin and Avastin, (6) Lenvatinib and Pembrolizumab, (7) Topotecan and Bevacizumab

Notably, the analyses of the specific tumour fraction through longitudinal samples allowed for the identification of the disease recurrence months before (4.68 ± 2.98) the clinical confirmation of relapse, mainly in patients with detectable pre-surgery ctDNA (80%, 8/10, Fig. 5A). However, in 4 (18.18%) of the 22 patients with recurrent disease, ctDNA was not detectable with the ddPCR approach (Supplementary Fig. 5G). Most of these patients were also ctDNA negative at surgery, showing the need to improve the sensitivity of the ctDNA detection to monitor low-shedding tumours. In addition, only 3 of the 37 samples analyzed one month after surgery were positive. Accordingly in these patients the surgery was not radical, and the presence of residual disease was known by the gynaecologists.

Importantly, longitudinal analyses of ctDNA proved to be a powerful tool to identify patients undergoing an early relapse, as reflected the patient #1 described in Fig. 5B. This patient was diagnosed with a FIGO stage IB endometrioid tumour that was positive for a pathogenic mutation in *FGFR2* in pre-surgical cfDNA (0.06%), which was found increased 5 months after surgery (4.52%) and clinically y confirmed two months later as an abdominal recurrence (18%). The patient started to receive Dostarlimab (a PD-1 blocker) and the ctDNA strongly decreased (0.38%) in line with the partial response defined based on CT-Scan. The patient is currently in response (0%) and is being monitored by means of the ctDNA along with the imaging.

Furthermore, longitudinal analyses also allow for the dynamic characterization of the disease in response to therapy pressure (Fig. 5C-D). For example, in Fig. 5C shows the case of a patient that was diagnosed with high-grade serous histology of EC and FIGO IIIA. NGS analyses of the UA showed alterations within the *PPP2R1A* and *TP53* genes and they were followed through the course of the disease. The patient had high levels of cfDNA at surgery as well as detectable levels of ctDNA. According to clinical guidelines the patient was treated with carboplatin-paclitaxel combination therapy and radiotherapy. Afterwards, the ctDNA levels were measured again and still detectable levels were found, indicating persistence of the disease (0.29%). Shortly thereafter, the patient was confirmed to have a liver recurrence by CT-scan, which showed a spike in ctDNA levels (16.90%). Following the relapse, the patient was treated with a second-line chemotherapy. Although there was an initial reduction on ctDNA levels, they started to increase again and the patient showed progressive disease with peritoneal affectation and entered PS-ECOG 4 and could no longer be treated.

Another example is shown in Fig. 5D, a patient diagnosed with a mixed histology phenotype (initially diagnosed as endometrioid), grade 2, FIGO IB tumour that showed high levels of cfDNA and ctDNA at surgery. After a year, patient showed symptoms compatible with a disease relapse at the lungs. At this moment ctDNA was positive confirming the recurrence of the disease. Due to the nature of the relapse the patient was closely monitored throughout the course of the disease, and the cfDNA/ctDNA kinetics reflect the evolution of the disease and the response to therapy, allowing the detection of disease recurrence prior to clinical evidence. Thanks to this approach, clinicians have been able to adjust the treatment and anticipate CT-scans according to the tumour kinetics. It is important to note that traditionally clinical variables identified this patient as being of intermediate risk, but our combinatory approach of cfDNA and ctDNA analyses classified it as of being of high-risk of recurrence, reinforcing the additional value of liquid biopsy to anticipate the disease relapse.

## Discussion

Liquid biopsy analyses have a great potential to improve the risk stratification and the follow up in the context of EC but data from larger and well characterized cohorts of patients are key to demonstrating their real clinical utility. With the present study we went a step further to define the value of liquid biopsy in EC and demonstrated the value of cfDNA/ctDNA as a prognostic and follow-up tool in a robust cohort of patients with localized disease recruited in a multicentric study.

Although the cfDNA origin is still unclear, several release pathways have been proposed, including apoptosis, necrosis and/or NETosis, or from extracellular vesicles, among others [30, 31]. The majority of plasma cfDNA found in healthy people is thought to be derived from nucleated blood cells, such as neutrophils and lymphocytes [30]. CfDNA levels are influenced by many factors such as age, metabolic activity, immune processes and diseases, such as cancer [32]. Compared to healthy people, patients with different solid tumour types have high cfDNA levels, which has been associated with poor prognosis in advanced stages [29, 33]. Appart from the contribution of the tumour derived cfDNA in this increment, the neoplasic transformation may have systemic effect on cell turnover or DNA clearance associated with the cfDNA dynamic [29]. In the present study we have shown that pre-surgery assessment of cfDNA levels correlates with poor clinical outcome, being a robust and independent prognostic biomarker for EC patients. We found a trend to have higher levels of cfDNA in high grade and myometrial/lymph vascular invasion as it was previously described by our group and other groups with different methodologies [21, 26, 34]. High cfDNA levels in high-risk EC patients can be partially explained by an increased in the ctDNA release but also other systemic mechanisms which are more intensely regulated in advanced stages of the disease. Although, our study did not reveal a correlation between cfDNA and the pre-surgery levels of different blood cells population, we could not rule out the impact of this population on the cfDNA content without the application of more specific analyses to characterize the cfDNA origin in our EC population. Nevertheless, data presented clearly pointed to the cfDNA analysis is a simple and cost-effective biomarker with prognostic value at surgery.

Indeed, when we specifically analyse the ctDNA fraction through personalized ddPCR assays to track MSI markers or pathogenic alterations (SNVs or CNVs) identified in the UAs, we found detectable levels in the 38% of the global cohort of patients, with this positivity being more frequent in high grade (41.10%) or deep infiltrating tumours (46.91%). Regarding the molecular EC subtypes, *TP53* mutant tumours also showed higher detection of pre-surgery ctDNA and VAFs were found higher in tumours with high-risk characteristics. These data are consistent with previous studies showing that high ctDNA content is associated with high-risk or advanced endometrial tumours [19, 21, 23, 26, 35]. Accordingly, we found that patients with detectable levels of ctDNA at surgery had significantly shorter DFS and DSS times, although with no independent value over the rest of clinical variables, probably due to the strong correlation with the other risk factors under study.

The combination of both high levels of cfDNA and detectable ctDNA data at surgery allowed for the identification of a group of patients that showed an extremely poor clinical outcome. Although this group represents the 10% of the global patient cohort analysed in our study, most of them developed an early relapse within the first year after surgery. The combined analysis of the pre-surgery cfDNA and ctDNA served to discriminate the risk of recurrence independently the histology, the FIGO stage, the grade or the molecular subtype. In fact, combined DNA analyses served to identify the patients that will recur within the groups of low, intermediate-high or high-risk tumours according to ESGO/ESTRO/ESP classification. It is important to mention that patients with low levels of cfDNA and detectable levels of ctDNA at surgery showed a poor evolution in comparison with the patients with low cfDNA and undetectable ctDNA. However, this group was not so clinically relevant in comparison with the high cfDNA and positive ctDNA. We hypothesize that high cfDNA levels are indicating local or systemic changes associated with the tumour aggressiveness, non-directly linked to the ctDNA release, but impacting on the disease biology.

Our data demonstrate the clinical interest of cfDNA/ctDNA analysis at surgery to improve the current risk stratification of patients with EC. In low and intermediate risk patients, although few cases were reclassified with the liquid biopsy approach, their DFS was significantly shorter. Outstandingly, the ctDNA assessment in patients with high cfDNA or other risk factors should be taken into consideration, as the presence of ctDNA presence will be a key tool in defining the patient cohort that would benefit either closer follow-up or intensification of the adjuvant treatment.

Notably, the ctDNA monitoring represents a valuable approach to detect the presence of minimal residual disease, anticipate relapse and evaluate the response to the therapy in advanced EC as the present study and other works have evidenced [21, 36, 37]. From the total cohort of patients included in the study, we monitored the cfDNA and ctDNA levels in longitudinal plasma samples from 130 patients. Globally, the cfDNA dynamic lacked value to accurately mirror the tumour burden although in specific cases cfDNA levels changed accordingly with the tumour evolution. This result could be explained by the addition of other factors such as the adjuvant therapy that can modify systemically the cfDNA release into circulation [38]. Of note, in the follow-up setting ctDNA monitoring showed high value to track the disease evolution as evidence the particular cases described. Post-surgery ctDNA was only detected in cases with residual or recurrent disease. Specially in cases with presence of pre-surgical ctDNA, the ctDNA kinetics served to detect the relapse months before the clinical/radiological confirmation, providing an opportunity to start the treatment earlier and accounting with the molecular information of the tumour clones that are driving the recurrent disease. The clinical relevance of post-surgery ctDNA monitoring has been well documented in other tumour types like colorectal, lung or breast tumours [39–42] and also in EC longitudinal ctDNA assessment by NGS and ddPCR has been successfully applied to detect the disease progression [21, 36, 37].

Our study robustly demonstrates the utility of ctDNA monitoring for more personalized and accurate disease follow-up. Despite these positive results, our approach has some limitations. ctDNA detection pre/post-surgery was not efficient in 20% of patients who relapsed. In this regard, it’s important to mention that our approach prioritises sensitivity by analysing the most predominant alterations found ineach patient´s primary tumour. Nonetheless, this may be related to the innate intratumoral heterogeneity described in EC [10]. Consequently, the predominant tumor clone responsible for the relapse may not be present in the bloodstream, and the emergence of new mutations during tumour evolution has not been considered. Furthermore, other technologies, such as panel-based strategies, would circumvent this limitation at the expense of sensitivity [21, 37]. Another study’s limitation is the lack of knowledge about the origin of the cfDNA found within the different sampling sites. No correlation was found between blood cell levels and the cfDNA dynamics, but this cannot rule out their contribution without some other type of molecular characterization.

## Conclusion

The present study demonstrates the value of cfDNA and ctDNA analyses as prognostic tools in the largest EC cohort published to date. High levels of cfDNA and detectable levels of ctDNA at surgery were strongly correlated with poor prognosis and served to identify the patients with early recurrence independent of other d EC risk factors. Additionally, longitudinal ctDNA assessment allowed early detection of recurrences and development of therapy resistance. Implementation of this approach in the clinic would lead to much better management of EC patients, reducing overtreatment and identifying patients at higher risk of recurrence for close monitoring. Although still early, our data suggest that the implementation of liquid biopsy in the clinic could significantly improve the management of the EC patients.

## Electronic supplementary material

Below is the link to the electronic supplementary material.

Supplementary Material 1

Supplementary Material 2

Supplementary Material 3

Supplementary Material 4

Supplementary Material 5

Supplementary Material 6

Supplementary Material 7

Supplementary Material 8

Supplementary Material 9

Supplementary Material 10

Supplementary Material 11

Supplementary Material 12

Supplementary Material 13

Supplementary Material 14

## Data Availability

The datasets used and/or analysed during the current study are available from the corresponding author on reasonable request.

## Abbreviations

cfDNA: Circulating free DNA
ctDNA: Circulating tumour DNA
EC: Endometrial Cancer
HCN: High Copy number
HR: Hazards Ratio
LVSI: Lymphovascular space invasion
MSI: Microsatellite Instability
NSMP: Non Specific Molecular Profile
DSS: Disease Specific Survival
DFS: Disease Free Survival
ROC: Receiver operator curve
TCGA: The Cancer Genome Atlas
UA: Uterine aspirate
VAF: Variant Allelic frequency
