# Supplementary figures and images for: Role of cfDNA and ctDNA to improve the risk stratification and the disease follow-up in patients with endometrial cancer: towards the clinical application
Source: J Exp Clin Cancer Res. 2024 Sep 20;43:264. doi: 10.1186/s13046-024-03158-w (PMC11414036; doi:10.1186/s13046-024-03158-w)

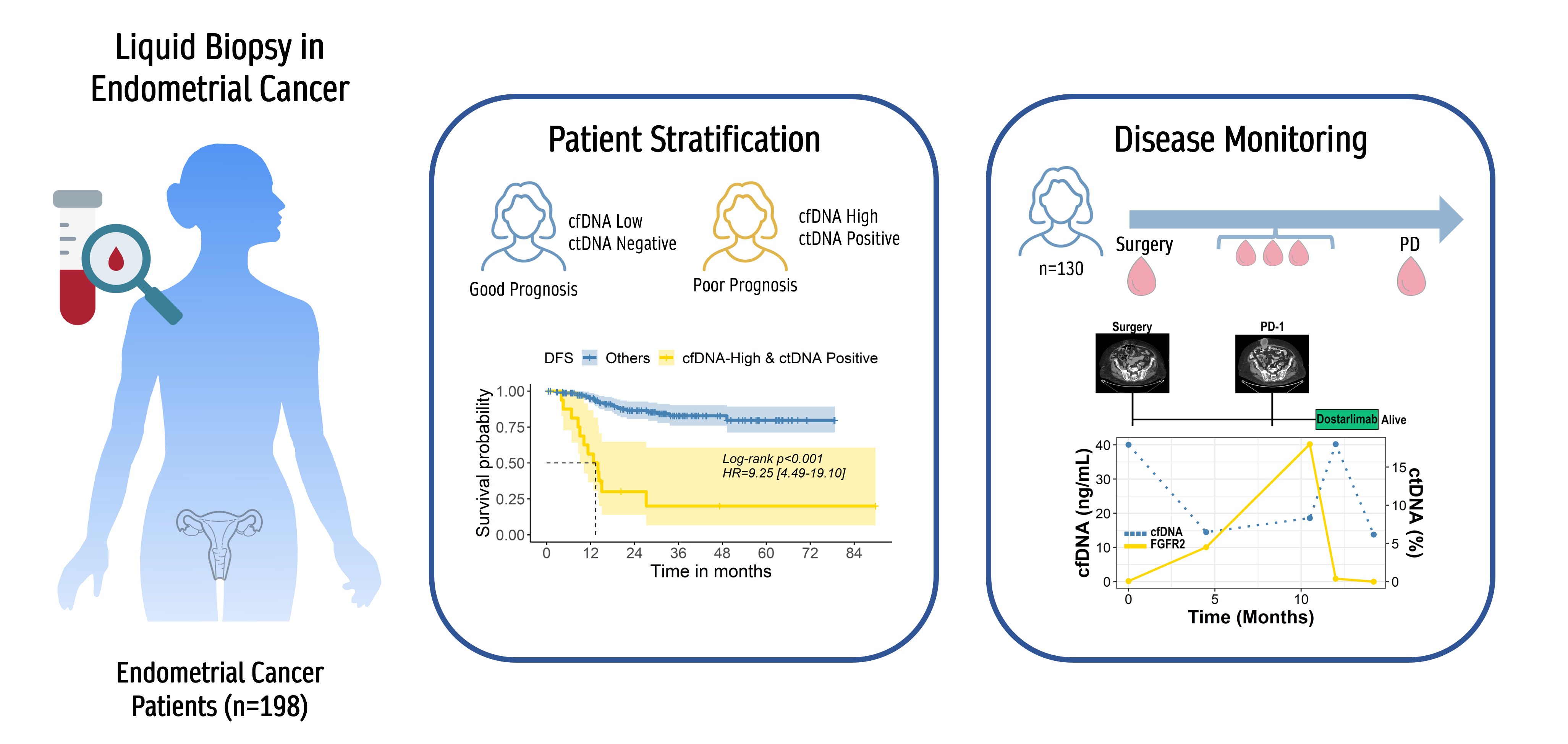

Supplement: Supplementary file 2 — Supplementary Material 2 [file 13046_2024_3158_MOESM2_ESM.tif]

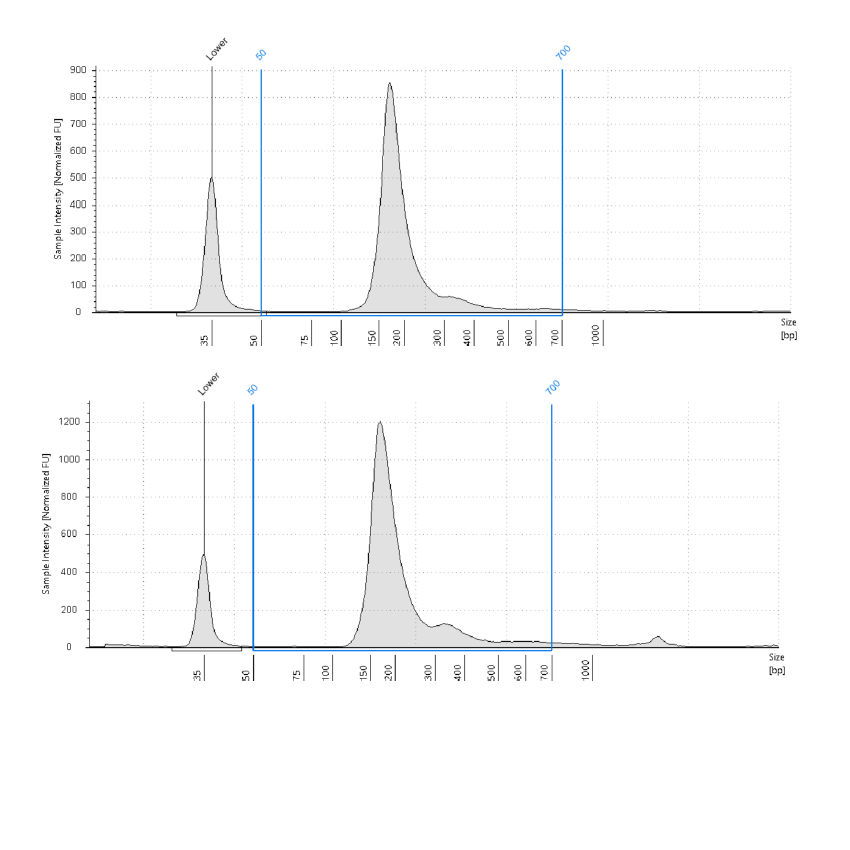

Supplement: Supplementary file 7 — Supplementary Material 7 [file 13046_2024_3158_MOESM7_ESM.tif]
