# Supplementary material for: Role of cfDNA and ctDNA to improve the risk stratification and the disease follow-up in patients with endometrial cancer: towards the clinical application
Source: J Exp Clin Cancer Res. 2024 Sep 20;43:264. doi: 10.1186/s13046-024-03158-w (PMC11414036; doi:10.1186/s13046-024-03158-w)
Supplement: Supplementary file 3 — Supplementary Material 3 [file 13046_2024_3158_MOESM3_ESM.docx]

**Supplementary Table 1.** Clinical characteristics of the studied cohort.

| **CLINICAL VARIABLES** | **N=198** | **CLINICAL VARIABLES** | **N=198** |
| --- | --- | --- | --- |
| **Age** | 181 | **Lymphovascular Infiltration** | 171 |
| Median (IQR) | 67 (58-73) | No | 137 (80%) |
| **Histology** | 196 | Yes | 34 (20%) |
| NEEC | 48 (24%) | **TCGA** | 194 |
| EEC | 148 (76%) | POLE | 15 (7.7%) |
| **Tumour Grade** | 197 | MSI | 76 (27%) |
| Grade 1 | 78 (40%) | NSMP | 53 (27%) |
| Grade 2 | 36 (18%) | HCN | 50 (26%) |
| Grade 3 | 83 (42%) | **cfDNA Concentration (ng/mL**) | 198 |
| **FIGO Stage** | 193 | Median (IQR) | 15 (10,24) |
| I | 126 (65%) | **ctDNA Positivity** | 177 |
| II | 29 (15%) | Detected | 52 (29%) |
| III | 29 (15%) | **Progression Diseas**e | 198 |
| IV | 9 (4.7%) | No | 161 (81%) |
| **Myometrial Infiltration** | 195 | Yes | 37 (19%) |
| <50% | 103 (53%) | **Death of Disease** | 198 |
| >50% | 92 (47%) | No | 174 (88%) |
|  |  | Yes | 24 (12%) |
