# Supplementary material for: Role of cfDNA and ctDNA to improve the risk stratification and the disease follow-up in patients with endometrial cancer: towards the clinical application
Source: J Exp Clin Cancer Res. 2024 Sep 20;43:264. doi: 10.1186/s13046-024-03158-w (PMC11414036; doi:10.1186/s13046-024-03158-w)
Supplement: Supplementary file 4 — Supplementary Material 4 [file 13046_2024_3158_MOESM4_ESM.docx]

**Supplementary Table 2.** Correlation between the presurgical cfDNA levels and ctDNA detection and different clinicopathologic features.

| **Variable** | | **cfDNA (ng/mL)^1^** | | **p-value** | | **ctDNA Positivity** | | **ctDNA VAF (%)^1^** | | **p-value** |
| --- | --- | --- | --- | --- | --- | --- | --- | --- | --- | --- |
| **Histology** |  | |  | |  | |  | |  | |
| EEC | | 15 (11-23) | | 0.5^2^ | | 28.15% (38/136) | | 0 (0-0.07) | | 0.3^2^ |
| NEEC | | 13 (9-25) | |  | | 35.00% (14/40) | | 0 (0-0.34) | |  |
| **Grade** |  | |  | |  | |  | |  | |
| G1-G2 | | 15 (11-23) | | >0.9^2^ | | 21.36% (22/103) | | 0 (0-0) | | **0.002**^2^ |
| G3 | | 15 (10-24) | |  | | 41.10% (30/73) | | 0 (0-0.44) | |  |
| **FIGO** |  | |  | |  | |  | |  | |
| I-II | | 15 (10-23) | | 0.3^2^ | | 23.18% (32/138) | | 0 (0-0) | | **<0.001**^2^ |
| III-IV | | 16 (11-26) | |  | | 52.95% (18/34) | | 0.04 (0-1.19) | |  |
| **Myometrial infiltration** |  | |  | |  | |  | |  | |
| <50% | | 13 (9-21) | | **0.005**^2^ | | 13.98% (13/93) | | 0 (0-0) | | **<0.001**^2^ |
| >50% | | 16 (12-24) | |  | | 46.92% (38/86) | | 0 (0-0.37) | |  |
| **LVSI** |  | |  | |  | |  | |  | |
| No | | 15 (10-23) | | **0.025**^2^ | | 18.85% (23/122) | | 0 (0-0) | | **<0.001**^2^ |
| Yes | | 20 (11,31) | |  | | 46.67% (16/30) | | 0.07 (0-0.42) | |  |
| **TCGA** |  | |  | |  | |  | |  | |
| POLE | | 15 (11-18) | | 0.6^3^ | | 17.67% (3/14) | | 0 (0-0) | | 0.10^3^ |
| MSI | | 15 (12-23) | |  | | 35.13% (26/74) | | 0 (0-0.16) | |  |
| NSMP | | 15 (11-21) | |  | | 17.78% (8/45) | | 0 (0-0) | |  |
| HCN | | 13 (8-25) | |  | | 37.50% (15/40) | | 0 (0-0.39) | |  |
| **Risk of recurrence** |  | |  | |  | |  | |  | |
| Low/intermediate low | | 14 (10-23) | | 0.2^2^ | | 16.67% (14/84) | | 0 (0-0) | | **<0.001**^2^ |
| High/high- intermediate | | 15 (11-23) | |  | | 40.86% (55/93) | | 0 (0-0.4) | |  |
| **Relapse** |  | |  | |  | |  | |  | |
| No | | 15 (10-21) | | **0.008**^2^ | | 22.22% (32/144) | | 0(0-0) | | **<0.001**^2^ |
| Yes | | 23 (11,31) | |  | | 60.61% (20/33) | | 0.12 (0-1.37) | |  |
| **Death of Disease** |  | |  | |  | |  | |  | |
| No | | 15 (10-21) | | **<0.001**^2^ | | 24.36% (38/156) | | 0 (0-0) | | **<0.001**^2^ |
| Yes | | 27 (17,44) | |  | | 66.67% (14/21) | | 0.22 (0-1.37) | |  |
| ^1^Median (IQR) | | | | | | | | | | |
| ^2^Wilcoxon rank sum test | | | | | | | | | | |
| ^3^Kruskal-Wallis rank sum test | | | | | | | | | | |
