# Supplementary material for: Role of cfDNA and ctDNA to improve the risk stratification and the disease follow-up in patients with endometrial cancer: towards the clinical application
Source: J Exp Clin Cancer Res. 2024 Sep 20;43:264. doi: 10.1186/s13046-024-03158-w (PMC11414036; doi:10.1186/s13046-024-03158-w)
Supplement: Supplementary file 5 — Supplementary Material 5 [file 13046_2024_3158_MOESM5_ESM.docx]

**Supplementary Table 3. cfDNA analyses identify the patients with the worst clinical outcome.** Cox proportional-hazards model was used to determine the relationship between clinical variables and the experimental variables.

| **Variable** | **Univariate** | | | | | **Multivariate** | | | |
| --- | --- | --- | --- | --- | --- | --- | --- | --- | --- |
|  | **N** | **HR***^1^* | **95% CI***^1^* | **p-value** | **q-value***^2^* | **HR***^1^* | **95% CI***^1^* | **p-value** | **q-value***^2^* |
| **Disease Free Survival** | | | | | | | | | |
| Histology | 196 | 3.72 | 1.95,7.11 | **<0.001** | **<0.001** | 2.16 | 0.83, 5.65 | 0.10 | 0.37 |
| Grade | 197 | 5.42 | 247,11.9 | **<0.001** | **<0.001** | 1.84 | 0.58, 5.81 | 0.30 | 0.37 |
| FIGO Stage | 193 | 4.04 | 2.10, 7.79 | **<0.001** | **<0.001** | 1.68 | 0.68, 4.17 | 0.26 | 0.37 |
| Myometrial Infiltration | 195 | 2.36 | 1.16, 4.77 | **0.013** | **0.015** | 1.31 | 0.49, 3.51 | 0.60 | 0.60 |
| LVSI | 171 | 4.26 | 2.17, 8,38 | **<0.001** | **<0.001** | 1.72 | 0.65, 4.55 | 0.28 | 0.37 |
| MSI Status | 175 | 0.62 | 0.30, 1.27 | 0.21 | 0.21 |  |  |  |  |
| TP53 Status | 188 | 4.08 | 1.90, 8.75 | **<0.001** | **<0.001** | 1.66 | 0.60, 4.57 | 0.32 | 0.37 |
| cfDNA Levels | 198 | 3.91 | 2.04, 7.51 | **<0.001** | **<0.001** | 2.98 | 1.35, 6.61 | **0.008** | **0.058** |
| **Disease Specific Survival** | | | | | | | | | |
| Histology | 196 | 4.46 | 1.95,10.2 | **<0.001** | **<0.001** | 1.05 | 0.34,3.32 | 0.93 | 0.93 |
| Grade | 197 | 16.4 | 3.84,70.2 | **<0.001** | **<0.001** | 4.75 | 0.85,26.5 | 0.056 | 0.13 |
| FIGO Stage | 193 | 7.10 | 3.07,16.4 | **<0.001** | **<0.001** | 4.35 | 1.30,14.6 | **0.015** | **0.054** |
| Myometrial Infiltration | 195 | 2.54 | 1.00,16.4 | **0.038** | **0.043** | 1.28 | 0.31,5.35 | 0.73 | 0.93 |
| LVSI | 171 | 4.90 | 2.07,11.6 | **<0.001** | **<0.001** | 0.86 | 0.23,3.25 | 0.83 | 0.93 |
| MSI Status | 175 | 0.52 | 0.20,1.35 | 0.16 | 0.16 |  |  |  |  |
| TP53 Status | 188 | 9.32 | 2.74,31.6 | **<0.001** | **<0.001** | 3.05 | 0.72,12.9 | 0.11 | 0.19 |
| cfDNA Levels | 198 | 6.54 | 2.83,15.1 | **<0.001** | **<0.001** | 9.13 | 2.82,29.5 | **0.001** | **0.001** |
| *^1^* HR = Hazard Ratio, CI = Confidence Interval | | | | | | | | | |
| *^2^* False discovery rate correction for multiple testing | | | | | | | | | |
