# Supplementary material for: Role of cfDNA and ctDNA to improve the risk stratification and the disease follow-up in patients with endometrial cancer: towards the clinical application
Source: J Exp Clin Cancer Res. 2024 Sep 20;43:264. doi: 10.1186/s13046-024-03158-w (PMC11414036; doi:10.1186/s13046-024-03158-w)
Supplement: Supplementary file 6 — Supplementary Material 6 [file 13046_2024_3158_MOESM6_ESM.docx]

**Supplementary Table 4. ctDNA analyses allow for the identification of the patients with the worst clinical outcome.** A cox proportional-hazards model was used to determine the relationship between clinical variables and the experimental variables.

| **Variable** | **Univariate** | | | | | **Multivariate** | | | |
| --- | --- | --- | --- | --- | --- | --- | --- | --- | --- |
|  | **N** | **HR***^1^* | **95% CI***^1^* | **p-value** | **q-value***^2^* | **HR***^1^* | **95% CI***^1^* | **p-value** | **q-value***^2^* |
| **Disease Free Survival** | | | | | | | | | |
| Histology | 196 | 3.72 | 1.95,7.11 | **<0.001** | **<0.001** | 2.57 | 0.99,6.69 | **0.047** | 0.16 |
| Grade | 197 | 5.42 | 247,11.9 | **<0.001** | **<0.001** | 1.26 | 0.37,4.25 | 0.71 | 0.75 |
| FIGO Stage | 193 | 4.04 | 2.10, 7.79 | **<0.001** | **<0.001** | 1.25 | 0.44,3.51 | 0.68 | 0.75 |
| Myometrial Infiltration | 195 | 2.36 | 1.16, 4.77 | **0.013** | **0.015** | 0.85 | 0.30,2.35 | 0.75 | 0.75 |
| LVSI | 171 | 4.26 | 2.17, 8,38 | **<0.001** | **<0.001** | 2.38 | 0.83,6.79 | 0.10 | 0.24 |
| MSI Status | 175 | 0.62 | 0.30, 1.27 | 0.21 | 0.21 |  |  |  |  |
| TP53 Status | 188 | 4.08 | 1.90, 8.75 | **<0.001** | **<0.001** | 1.33 | 0.45,3.99 | 0.61 | 0.75 |
| ctDNA Levels | 177 | 3.63 | 1.80,7.30 | **<0.001** | **<0.001** | 2.70 | 1.12,6.46 | **0.025** | 0.16 |
| **Disease Specific Survival** | | | | | | | | | |
| Histology | 196 | 4.46 | 1.95,10.2 | **<0.001** | **<0.001** | 2.11 | 0.71,6.31 | 0.18 | 0.35 |
| Grade | 197 | 16.4 | 3.84,70.2 | **<0.001** | **<0.001** | 2.56 | 0.39,16.9 | 0.31 | 0.39 |
| FIGO Stage | 193 | 7.10 | 3.07,16.4 | **<0.001** | **<0.001** | 2.25 | 0.66,7.65 | 0.19 | 0.35 |
| Myometrial Infiltration | 195 | 2.54 | 1.00,16.4 | **0.038** | **0.043** | 0.55 | 0.14,2.15 | 0.39 | 0.39 |
| LVSI | 171 | 4.90 | 2.07,11.6 | **<0.001** | **<0.001** | 2.32 | 0.62,8.72 | 0.20 | 0.35 |
| MSI Status | 175 | 0.52 | 0.20,1.35 | 0.16 | 0.16 |  |  |  |  |
| TP53 Status | 188 | 9.32 | 2.74,31.6 | **<0.001** | **<0.001** | 2.07 | 0.45,9.58 | 0.33 | 0.39 |
| ctDNA Levels | 177 | 3.91 | 1.57,9.74 | **0.002** | **0.003** | 3.40 | 1.18,9.77 | **0.018** | 0.13 |
| *^1^* HR = Hazard Ratio, CI = Confidence Interval | | | | | | | | | |
| *^2^* False discovery rate correction for multiple testing | | | | | | | | | |
