# Supplementary material for: Role of cfDNA and ctDNA to improve the risk stratification and the disease follow-up in patients with endometrial cancer: towards the clinical application
Source: J Exp Clin Cancer Res. 2024 Sep 20;43:264. doi: 10.1186/s13046-024-03158-w (PMC11414036; doi:10.1186/s13046-024-03158-w)
Supplement: Supplementary file 8 — Supplementary Material 8 [file 13046_2024_3158_MOESM8_ESM.docx]

**Supplementary Table 5.** Clinical characteristics of the cohort when divided based on the different combination groups.

| **Variable** | **N** | **Group 1** N=104 | **Group 2** N=36 | **Group 3** N=21 | **Group 4** N=16 | **p-value^2^** |
| --- | --- | --- | --- | --- | --- | --- |
| **Age** | 160 |  |  |  |  | 0.60 |
| Median [IQR] |  | 66 [56,73] | 69 [57,73] | 67 [62,75] | 69 [59,78] |  |
| **Histology** | 175 |  |  |  |  | 0.20 |
| Endometrioid |  | 79 (77%) | 30 (83%) | 18 (86%) | 8 (50%) |  |
| Serous |  | 16 (16%) | 4 (11%) | 2 (9.5%) | 8 (50%) |  |
| Clear cell |  | 3 (2.9%) | 0 (0%) | 0 (0%) | 0 (0%) |  |
| Mixed |  | 3 (2.9%) | 1 (3.0%) | 1 (4.8%) | 0 (0%) |  |
| Carcinosarcoma |  | 1 (1%) | 1 (2.8%) | 0 (0%) | 0 (0%) |  |
| **Tumour Grade** | 176 |  |  |  |  | **<0.001** |
| 1 |  | 53 (51%) | 7 (19%) | 9 (43%) | 0 (0%) |  |
| 2 |  | 13 (13%) | 11 (31%) | 6 (29%) | 4 (25%) |  |
| 3 |  | 37 (36%) | 18 (50%) | 6 (29%) | 12 (75%) |  |
| **FIGO Stage** | 172 |  |  |  |  |  |
| I |  | 76 (75%) | 15 (44%) | 17 (81%) | 5 (31%) |  |
| II |  | 11 (11%) | 10 (29%) | 3 (14%) | 2 (13%) |  |
| III |  | 12 (12%) | 7 (21%) | 1 (4.8%) | 6 (38%) |  |
| IV |  | 2 (2%) | 2 (5.9%) | 0 (0%) | 3 (19%) |  |
| **LVSI** | 152 |  |  |  |  | **<0.001** |
| No |  |  |  |  |  |  |
| Yes |  | 9 (9.7%) | 10 (38%) | 5 (25%) | 6 (46%) |  |
| **Myometrial Infiltration** | 174 |  |  |  |  | **<0.001** |
| <50% |  | 69 (67%) | 7 (20%) | 11 (55%) | 6 (38%) |  |
| >50% |  | 34 (33%) | 28 (80%) | 9 (45%) | 10 (63%) |  |
| **TCGA Classification** | 173 |  |  |  |  |  |
| POLE |  | 9 (8.80%) | 3 (8.30%) | 2 (11%) | 0 (0%) |  |
| MSI |  | 41 (40%) | 19 (53%) | 7 (37%) | 7 (44%) |  |
| NSMP |  | 31 (30%) | 7 (19%) | 6 (32%) | 1 (6.3%) |  |
| HCN |  | 21 (21%) | 7 (19%) | 4 (21%) | 8 (50%) |  |
| **cfDNA Concentration** | 177 |  |  |  |  | **<0.001** |
| Median [IQR] |  | 13 [9,16] | 15 [12,19] | 40 [28,56] | 31 [28,45] |  |
| **ctDNA Positivity** | 177 |  |  |  |  | **<0.001** |
|  |  | 0 (0%) | 36 (100%) | 0 (0%) | 16 (100%) |  |
| **Progression Disease** | 177 |  |  |  |  | **<0.001** |
| No |  | 95 (91%) | 28 (78%) | 17 (81%) | 4 (25%) |  |
| Yes |  | 9 (8.7%) | 8 (22%) | 4 (19%) | 12 (75%) |  |
| **Death of Disease** | 177 |  |  |  |  | **<0.001** |
| Alive |  | 100 (96%) | 32 (89%) | 18 (86%) | 5 (31%) |  |
| Dead |  | 4 (3.8%) | 4 (11%) | 3 (14%) | 11 (69%) |  |
| ^1^n (%) | | | | | | |
| ^2^Kruskal-Wallis rank sum test; Fisher’s exact test; Pearson’s Chi-squared test | | | | | | |
