# Supplementary material for: Role of cfDNA and ctDNA to improve the risk stratification and the disease follow-up in patients with endometrial cancer: towards the clinical application
Source: J Exp Clin Cancer Res. 2024 Sep 20;43:264. doi: 10.1186/s13046-024-03158-w (PMC11414036; doi:10.1186/s13046-024-03158-w)
Supplement: Supplementary file 14 — Supplementary Material 14 [file 13046_2024_3158_MOESM14_ESM.docx]

**Supplementary Figure 1: Pre-surgery cfDNA and ctDNA levels showed independence from other blood parameters.** **A-C** Spearman Correlation of the cfDNA levels with CA-125 (n=36) (**A**), CEA (n=18) (**B**) or PBMCs (n=58) levels (**C**)**. D-G** Violin plots showing tumour length (cm) according to the cfDNA levels (**D**) and ctDNA presence (**F**) and the tumour volume (cm^2^) according the cfDNA levels (**E**) and ctDNA presence (**G**). Statistical significance was evaluated by Mann–Whitney U. ns p-value>0.05.

**Supplementary Figure 2: Association between cfDNA and ctDNA. A** Violin plot of the cfDNA concentration versus the presence of ctDNA. **B** Violin plot of the cfDNA input used in the ddPCR according to the presence of ctDNA. **C** Spearman correlation between the cfDNA levels at baseline (ng/mL) and the ctDNA VAFs (%). **D** Spearman correlation between ctDNA levels (VAF=%) and cfDNA input used in the ddPCR.

**Supplementary Figure 3: Survival analyses according to pre-surgery cfDNA and ctDNA levels. A-B** Kaplan-Meier curves showing DFS (**A**) and DSS (**B**) in patients according to the pre-surgery levels of cfDNA and ctDNA. Group 1: low presurgical cfDNA levels and negative ctDNA, Group 2: low presurgical cfDNA levels and positive ctDNA levels, Group 3: high presurgical cfDNA levels and negative ctDNA, Group 4: high presurgical cfDNA levels and detectable ctDNA levels. **C-F** Kaplan-Meier curves showing DFS in patients according to the pre-surgery high levels of cfDNA and detectable levels of ctDNA in patients with EEC histology (**C**), NEEC histology (**D**), low grade (G1 or G2) (**E**), or high grade (G3) (**F**). **G-H** Kaplan-Meier curves showing DSS in patients according to the pre-surgery high levels of cfDNA and detectable levels of ctDNA in patients with low/intermediate (**G**) or high-intermediate/high (**H**) risk of recurrence according to the ESGO stratification.

**Supplementary Figure 4: Survival analyses according to the cfDNA, ctDNA and ESGO stratification risk. A-B** Kaplan-Meier curves showing DFS (**A**) and DSS (**B**) in patients according to the pre-surgery levels of cfDNA and ctDNA and the risk of recurrence according to the ESGO stratification. Good prognosis is defined as patients with low or intermediate risk of recurrence according to the ESGO stratification and negative levels of ctDNA. Poor prognosis is defined as patients with either high-intermediate/high risk of recurrence according to the ESGO stratification or high levels of cfDNA and detectable levels of ctDNA regardless of recurrence risk.

**Supplementary Figure 5: Longitudinal cfDNA follow-up. A-F** Violin plots of the cfDNA levels within the entire population (**A-F**) when compared between baseline and different time points: (**A**) 1-month post-surgery, (**B**) 6 months post-surgery, (**C**) 12 months post-surgery, (**D**) 18 months post-surgery, (**E**) 24 months post-surgery and (**F**) at disease progression. **G** Swimmer plot of all patients who experienced tumour progression divided based on the combinatorial approach. Wilcoxon signed-rank test was used to assess statistical significance between paired samples *p<0.05.

# **TABLE LEGENDS**

**Supplementary Table 1.** **Clinical characteristics of the studied cohort.**

**Supplementary Table 2. Correlation between the presurgical cfDNA levels and ctDNA detection and different clinicopathologic features.**

**Supplementary Table 3. cfDNA analyses identify the patients with the worst clinical outcome.** Cox proportional-hazards model was used to determine the relationship between clinical variables and the experimental variables.

**Supplementary Table 4. ctDNA analyses allow for the identification of the patients with the worst clinical outcome.** A cox proportional-hazards model was used to determine the relationship between clinical variables and the experimental variables.

**Supplementary Table 5.** **Clinical characteristics of the cohort when divided based on the different combination groups.**

**Supplementary Table 6. List of genomic alteration identified in the UAs using the Oncomine comprehensive Panel or MSI testing.** Variants monitored in cfDNA by are highlighted in bold.
